# Synthetic Biology: Environmental Health Implications of a New Field

**DOI:** 10.1289/ehp.118-a118

**Published:** 2010-03

**Authors:** Charles W. Schmidt

**Affiliations:** **Charles W. Schmidt**, MS, an award-winning science writer from Portland, ME, has written for *Discover Magazine*, *Science*, and *Nature Medicine*

Imagine the most sophisticated engineering feat you can think of, and you might not consider a living cell. And yet cells are fabulously sophisticated, able to produce all the proteins, tissues, and biological circuits that give rise to life. Scientists have spent hundreds of years just trying to understand cells and to work with them as they were created by nature. Now it’s becoming possible to “rewire” cells using genetic circuits, protein pathways, and other biomolecular machinery created in the laboratory. By swapping out natural genetic circuitry for synthesized components made of DNA, scientists are putting cells to work as sensors and as miniature factories that make pharmaceuticals, fuels, and industrial chemicals.

These possibilities not only blur the lines between engineering and biology but also are transforming how scientists approach challenges in energy, human health, and the environment. Robert Kitney, a professor of biomedical systems engineering at Imperial College of Science, Technology, and Medicine in London, England, believes the field’s influence could rival or exceed that of synthetic chemistry, which made modern pharmaceuticals, detergents, plastics, and computer semiconductors possible. “We’re talking about harnessing cells—which I describe as the ultimate manufacturing units—to carry out human-controlled processes,” says Kitney. “And that’s a completely new world with many up sides.”

David Rejeski, who directs the Science and Technology Innovation Program at the Woodrow Wilson International Center for Scholars in Washington, DC, predicts a steady convergence of nanotechnology and synthetic biology will redefine manufacturing over the next 100 years. “It’s a profound change—the next Industrial Revolution,” he says. “Precision control of matter at the nanoscale will change the way we produce just about everything, from electronics to drugs, fuels, materials, and food.”

## Defining the Field

Despite that potential—or perhaps because of it—this new field of synthetic biology suffers from an identity crisis. Ask 10 experts to define “synthetic biology,” and you’re liable to get 10 different answers. The field overlaps with genetic engineering, which involves adding or deleting single genes, and also with metabolic engineering, which allows scientists to optimize cellular processes to produce desired substances, such as hormones. Pamela Silver, a professor of systems biology at Harvard Medical School and a core faculty member with Harvard University’s Wyss Institute for Biologically Inspired Engineering, says synthetic biology embraces metabolic engineering but also diverges from it by relying on modular components made from DNA. Scientists can now synthesize genes from DNA subunits arranged to user specifications. Those genes are then strung together into components and devices that cells, under laboratory conditions, can absorb into their chromosomes.

The field of synthetic biology was launched by a pair of papers published in the 20 January 2000 issue of **Nature**. The first—by Michael B. Elowitz and Stanislas Leibler—presented a synthetic genetic oscillator. The other—by Timothy S. Gardner, Charles R. Cantor, and James J. Collins—presented a synthetic genetic toggle switch, showing that it was feasible to model, design, and construct synthetic gene networks out of biomolecular components.

In what’s seen as a major proof of concept for the field, scientists at Amyris Biotechnologies in Emeryville, California, rewired 12 genes in yeast so the organism would produce artemisinin, an antimalarial drug. On the environmental front, scientists are also rewiring algae and other organisms to make biofuels for the transportation sector. Eric Toone, a professor of chemistry and of biochemistry at Duke University, says that without synthetic biology it’s unlikely biofuels could ever be produced at the volumes and prices needed to compete economically with gasoline, diesel, or jet fuel.

But if synthetic biology is exciting, it’s also unsettling to those concerned about its risks. Engineered microbes might escape and propagate in the wild with unforeseen consequences, some say. Others caution that synthetic biology has high potential for abuse. Customized DNA sequences delivered through the mail can now be bought for just 40¢ per base pair. Gene synthesis companies aren’t legally obligated to screen their customers, so it’s possible terrorists could make viral bioweapons from scratch, says Pat Mooney, executive director with the ETC Group in Ottawa, Canada.

Jay Keasling, a professor of chemical engineering at the University of California, Berkeley, who pioneered the artemisinin research, openly acknowledges the field’s potential hazards. “The worst thing that could happen is someone gets hurt from synthetic biology,” he says. “But we’re also talking about applications that justify the field going forward in a major way.” Like other proponents of the field, Keasling frames synthetic biology’s potential in terms of how it can help solve humanity’s worst problems, many of which are tightly intertwined with environmental health: energy shortages, pollution, hunger, and disease.

“We’re headed towards a global population of nine billion in just thirty-five years, up from six billion today,” adds Craig Venter, who famously led private efforts to decode the human genome, and who now heads the J. Craig Venter Institute, a genomics-based research organization. “Our . . . hope is that [synthetic biology] works so that we don’t have to constantly destroy the environment to produce more food. The same applies to fuel—we need intelligent solutions.”

## A Focus on Biofuels

Given pricing, security, and pollution concerns regarding fossil fuels, biofuels rank high as a priority use for synthetic biology; figures collected by Rejeski’s team show the Department of Energy spent over $305 million on synthetic biology research in fiscal year 2009 with a similar amount projected for this year. (By comparison, the Department of Health and Human Services spent roughly $19 million in the field in fiscal year 2009 and has yet to determine its 2010 outlay.)

Unlike fossil fuels, which release long-sequestered carbon dioxide (CO_2_) into the atmosphere when burned, plant-based biofuels are carbon-neutral, meaning the carbon they release during burning was captured from the air during photosynthesis. The first-generation fuels available now—namely, corn-based ethanol, biodiesel, and other fuels derived from food crops—have been impractical as energy sources, Toone says. Ethanol is corrosive and miscible with water, so it can’t be transported by pipeline. And biodiesel can’t burn in gasoline engines, which power most of the vehicles on the road. What’s more, first-generation fuels are linked to instabilities in food pricing and also with deforestation in tropical countries [for more information, see “Food vs. Fuel: Diversion of Crops Could Cause More Hunger,” *EHP* 116:A254–A257 (2008)].

Next-generation biofuels generated from nonfood sources such as algae, cyanobacteria, and switchgrass—a weedy plant that grows on marginal lands, generating enormous biomass without much water—will ideally be produced more efficiently, relieving some pressure on agriculture. Scientists are engineering cells that secrete fuel intermediates (such as lipids and fatty acids) that can be refined into fuels. This past July, ExxonMobil contributed $600 million to Venter’s new startup company, Synthetic Genomics, Inc., with the aim of extracting “biocrude” from photosynthetic algae that can be refined into gasoline, diesel, and jet fuel.

Venter’s approach draws on the concept of making biofuel directly from CO_2_ in the atmosphere. Photosynthetic organisms such as algae fix CO_2_ from the air; then, using light (as an energy source) and hydrogen from water vapor, they reduce this CO_2_ to an energy-rich product: glucose. A sugar, glucose is loaded with carbon–carbon bonds. And during respiration, those bonds are broken down into lipids and other energy-rich hydrocarbons that could ideally be refined into transportation fuel.

By changing the algae’s genetic structure, Venter and his colleagues aim to make different types of hydrocarbons, more like those found in fossil fuels. Given proprietary concerns, Venter won’t comment on how his company is rewiring the algae. He says only that they’re “engineered to continuously pump hydrocarbons out into media [rather than accumulating them], making them production machines rather than something we grow just to kill or harvest.”

James Liao, a professor of chemical and biomolecular engineering at the University of California, Los Angeles, hopes to avoid refining altogether by engineering photosynthetic cyanobacteria that make engine-compatible fuels. As described in the December 2009 issue of *Nature Biotechnology*, Liao and colleagues divert cell pathways normally involved in amino acid synthesis so that instead they produce alcohol—namely, butanol, which Liao says can go directly into current-day internal combustion engines. “The good thing about algae and cyanobacteria is that they don’t require agricultural land,” Liao adds. “We can use coastal areas.”

Writing in the same issue of *Nature Biotechnology*, John Sheehan, a scientific program coordinator at the Institute on the Environment at the University of Minnesota, described Liao’s production volumes as “impressive,” pointing out they’re “five to six times better than industrially relevant estimates for corn and cellulosic ethanol production, and even outperform current estimates for algal oil productivity.”

Still, Liao acknowledges that even with these high yields, photosynthetic microbes would have to be cultivated on millions of acres to offset gasoline and other liquid fossil fuels. That’s in part because photons penetrate just 10 cm into the ponds and bioreactors where the microbes are grown.

Toone, who directs biofuels research at the Department of Energy’s Advanced Research Projects Agency—Energy (more commonly known as ARPA-E), agrees that biofuels derived from photosynthesis will require enormous land area regardless of whether energy crops or microbes are used. “And that brings us to another option that hasn’t been explored yet: using nonphotosynthetic organisms to make liquid fuels from carbon dioxide,” Toone says.

For those who aren’t familiar with synthetic biology, the term can conjure images of scientists creating artificial life—monsters, perhaps—in the laboratory. Newspaper headlines can feed those perceptions—a 2008 report by th Woodrow Wilson International Center for Scholars, *Trends in American and European Press Coverage of Synthetic Biology: Tracking the Last Five Years of Coverage*, found numerous media references to “playing God” or “copying God,” and even the phrase “Frankenstein-like” to describe what’s emerging from the field. The reality isn’t quite so sensational; scientists aren’t creating new life from scratch so much as they are developing new ways to direct cell behavior.

Nonphotosynthetic microbes take energy from sources other than light, such as charged ions in certain metals. But like their photosynthetic counterparts, these organisms don’t produce traditional fuel compounds—acetogenic microbes, for instance, make acetate during respiration, while methanogens produce methane. “We need synthetic biology to install new pathways so that these organisms start producing the fuels we’re interested in,” Toone says. “The bugs could go anywhere, even underground, and you don’t have to spread them so thinly because they don’t [rely on] photons.”

Robert Kelly, director of the biotechnology program at North Carolina State University, suggests that energy for nonphotosynthetic organisms could come from hydrogen, which some anaerobic microbes use to reduce CO_2_ into more complex carbon-based molecules. Toone adds that some microbes could be engineered to use electricity as an energy source. “You could generate that electricity from solar panels, nuclear power, even wind and wave action,” he says.

None of the bewildering array of options for making next-generation fuels are ready for prime time yet. And those deemed most promising will also have to contend with three core challenges, according to James Collins, a professor of bioengineering at Boston University and a Wyss Institute core member. “The first [challenge] is scale—you need to get production up to industrial levels,” Collins says. “The second is efficiency, because as the size of your operation grows, your fuel yield will likely decrease. And the third is economics. You can’t expect a viable business model if it costs you four dollars to make a dollar’s worth of gas. Failure to overcome any one of these limitations is likely to kill your project.”

Robert Carlson, a principal at Biodesic, a bioengineering design firm in Seattle, Washington, doesn’t necessarily see scale as a dealbreaker when it comes to commercializing biofuel applications. On the contrary, he wrote in a 23 February 2009 essay titled “The New Biofactories,” synthetic biology could enable the production of fuel within cars themselves: “In the spring of 2007, researchers reported the successful construction of a synthetic pathway consisting of 13 enzymes from different organisms that can turn starch into hydrogen,” he wrote. “This suggests a future in which sugar or starch—substances available at any grocery store—will go into our fuel tanks instead of gasoline.”

Kelly adds that no one approach is likely to serve as a silver bullet that replaces fossil fuels altogether. “We’re not going to be locked into any one system,” he says.

## Synthetic Microbes for Bioremediation

Apart from new fuels, better hazardous waste cleanup is also cited as one of synthetic biology’s environmental promises. Bioremediation is already common in oil spill cleanups; *Rhodococcus* and *Pseudomonas* bacteria, among others, naturally consume and degrade many petroleum components into less toxic by-products. Using engineered microbes to degrade more recalcitrant chemicals such as dioxins, pesticides, or even radioactive compounds could save millions of dollars otherwise spent on excavating and trucking polluted soils to hazardous waste landfills, according to Gary Sayler, who heads the Center for Environmental Biotechnology at the University of Tennessee in Knoxville. But research in this area—under development for more than 2 decades—has yet to get out of the laboratory, Sayler says. Fearing uncertain environmental consequences, activists have routinely lined up against releasing engineered microbes for cleanup, and the U.S. Environmental Protection Agency (EPA) has subjected the organisms to extensive risk-assessment protocols.

Today, health agencies are more willing to consider genetically engineered microbes in cleanup, Sayler says, but even so, the infrastracture needed to proceed isn’t available, and neither is the funding. Synthetic biology might offer new opportunities, he adds, but scientists need to explore how degradation pathways developed mainly in *Escherichia coli* research will work in other microbes better suited for survival in polluted sites.

A leading scientist in this area is Victor de Lorenzo, head of the Laboratory of Environmental Molecular Microbiology at Spain’s National Center for Biotechnology. De Lorenzo uses robust microbes that survive in harsh conditions—for instance, the soil bacterium *Pseudomonas putida*—which he then “edits” genomically by replacing nonessential genes with engineered metabolic and regulatory circuits that degrade target compounds. These new circuits direct microbes away from easy carbon sources such as glucose, he says, and toward more challenging food sources in industrial chemicals. “In other words, we’re uncoupling metabolism from the microbe’s own physiology,” he explains.

By removing all nonessential genes, de Lorenzo can create what’s known as a reduced genome, or a minimized cell. As blank slates that scientists can program by adding new genes, these constructs define a leading edge for synthetic biology.

Apart from making minimal cells by deleting unnecessary genes, scientists can also generate them by booting up voided cells (whose own chromosomes have been removed) with entirely new genomes assembled from scratch. This is the approach Venter is taking now. In 2008, he and his research team accomplished one of synthetic biology’s biggest feats: they synthesized the entire genome—485 coding genes—for *Mycoplasma genitalium*, a simple bacterium. According to Venter, at least 115 of those genes are nonessential and can be deleted without harming the genome’s functionality. Venter’s team is now trying to use a synthetic bacterial genome to boot up the voided cell of a related species, *M. capricolum*.

So far, as reported in the 25 September 2009 issue of *Science*, they haven’t succeeded. Venter explains that *M. capricolum* rejected the new genome in much the same way it might reject a virus. “We’re developing methods to sidestep this problem,” he says. Among those methods: removing the restriction enzymes that *M. capricolum* uses to slice up foreign genetic material (which led to the recent failure) or attaching methyl groups to the synthetic genome to protect it in the cell. If successful, Venter and his colleagues will produce a minimal cell possessing only the genes needed for life.

Whether such a cell would constitute a synthetic life form—as some have claimed—is up for debate, however. Petra Schwille, a professor at the Biotechnology Center of the Dresden University of Technology, says Venter’s microbe isn’t synthetic life so much as something more analogous to an interspecies clone. “He’s inserting the genome from one organism into the chassis of another,” she explains. That’s different from synthesizing an entire living cell from fatty acids and proteins. To me, this is more like a bacterial robot than a type of synthetic life.”

Venter emphasizes that his ambition in making this type of cell has always been to use them as platforms for understanding fundamental living processes. Still, Silver emphasizes that regardless of how they’re made, minimal cells could also be used as basic manufacturing platforms. Just like a computer’s functionality depends on the software you put into it, she explains, a minimal cell’s functionality would depend on its synthetic circuits. “If you want to make fuel or drugs, you still use this as your platform organism,” she says. “It’s essentially a universal chassis onto which you layer everything else.”

## Regulating the Future

Meanwhile, experts disagree on how risky any of these engineered microbes might be. Keasling argues they don’t compete well in the wild and, moreover, that scientists can engineer the organisms to die when their task is complete—for instance, after the nutrient pollutants they feed on run out. And Collins has created DNA counters that commit cells to death after they replicate a few times.

Still, as Rejeski wrote in the January/February 2010 issue of *The Environmental Forum*, “One important lesson from the last Industrial Revolution is that the winners in this technological arms race are not necessarily good for the environment.” Synthetic biology promises a galaxy of molecules and systems that are “specifically engineered to respond to the external environment (for instance, change structure and behavior in response to light, electromagnetic fields, pH, or other conditions), or actually self-assemble into entirely new structures,” he wrote. “These applications will be difficult to understand with traditional risk assessment methods.”

Kitney says the interface between gene synthesis companies and the public will ultimately form the front line for new regulations. “Right now, the research community in this area is pretty small,” he says. “But as it gets larger—and I completely believe that it will—we’ll need to go from voluntary systems to more rigorous regulations that monitor potential threats.”

Mooney also cautions that biofuel development could still compete unacceptably for agricultural resources and consolidate intellectual property in fuels and manufacturing in the hands of just a few companies. The ETC Group’s October 2008 report *Commodifying Nature’s Last Straw? Extreme Genetic Engineering and the Post-Petroleum Sugar Economy* states, “Advocates of synthetic biology and the bio-based sugar economy assume that unlimited supplies of cellulosic biomass will be available. But can massive quantities of biomass be harvested sustainably without eroding/degrading soils, destroying biodiversity, increasing food insecurity and displacing marginalized peoples?” Moreover, the report states, simply “moving beyond petroleum” does not address high consumption patterns that drive many of these environmental ills.

In Mooney’s view, the regulations governing synthetic biology now are wholly inadequate. That’s not to say the risks outweigh the potential benefits, he emphasizes. “We’re not talking about a failure of science but of governance in terms of its ability to track and regulate a powerful new technology,” he explains. “This capacity to redesign life is vastly greater than what we normally associate with biotechnology.”

As Rejeski put it in his *Environmental Forum* essay, “EPA and the other environmental agencies have a once-in-a-century opportunity to place environmental policy and protection in front of a major shift in how we produce just about everything.” What’s needed, Rejeski says, is a central authority that coordinates research and planning on synthetic biology. An analogous entity, he says, might be the National Nanotechnology Coordination Office, which organizes federal research and development, public information, and congressional hearings into that field. “There’s not enough public engagement on the science of synthetic biology or its social and ethical implications, but from what we can tell in our focus groups and surveys, this is going to be a really contentious issue,” Rejeski says. “People react very negatively to the phrase ‘synthetic biology,’ and it’s going to be hard to thread the science through the needle of public opinion.”

Still, Mooney lauds what he says is a remarkably open dialogue between scientists and policy experts in the technology’s early days. “It can’t just be scientists making all the decisions here,” he says. “We also need governments that represent the people, who can talk to the scientists and beyond them. I think if people have the chance to think these things through carefully, we’ll end up saying no in some cases, but in others, we’re going to want to know how we can use [synthetic biology] to solve problems.”

## Figures and Tables

**Figure:**
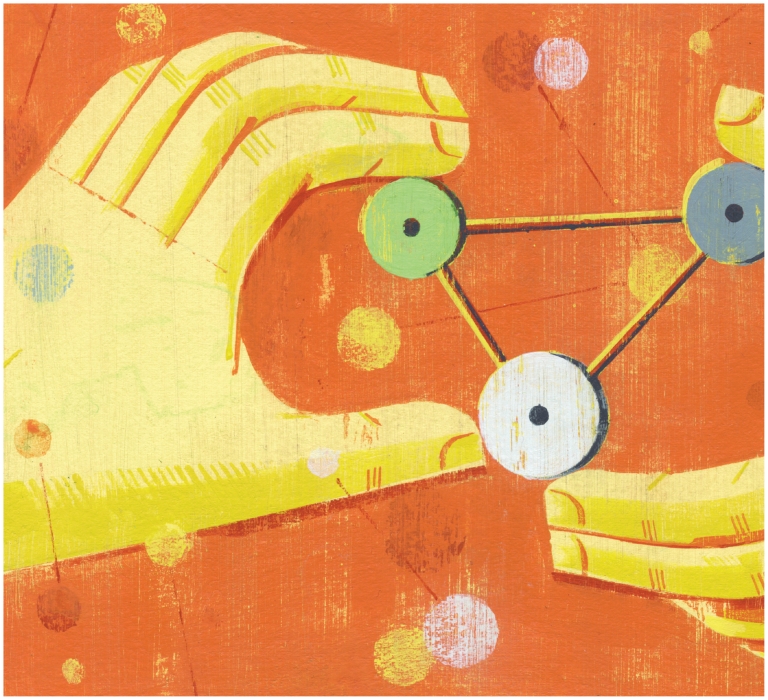


**Figure:**
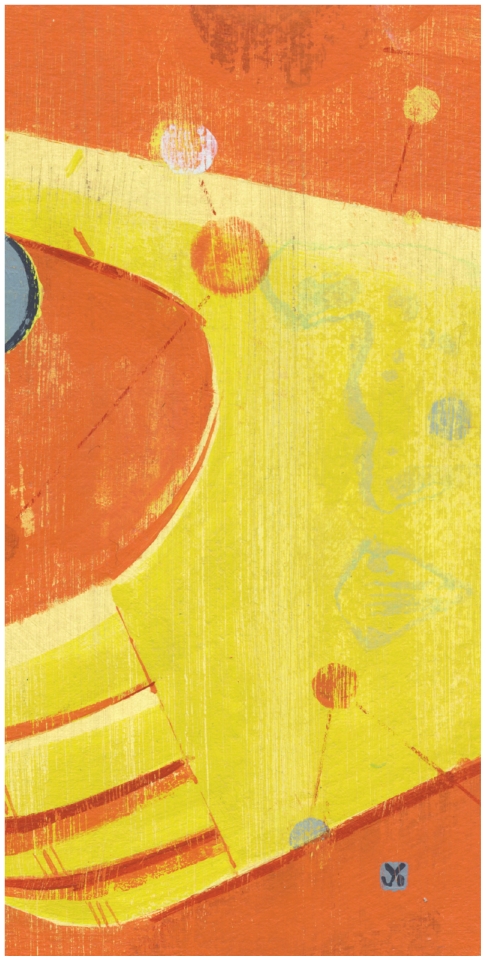


**Figure:**
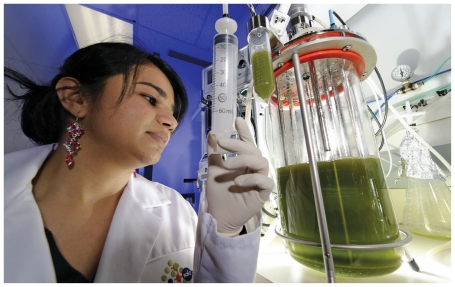
Researchers currently are aiming to produce 100 g of biofuel per m^2^ per day from algae and cyanobacteria—about 10 times the output achieved so far. Equaling current U.S. demand for gasoline would require millions of acres if using photosynthetic algae, but novel strains of nonphotosynthetic algae can be grown in fermentation bioreactors that could require less land area.

**Figure:**
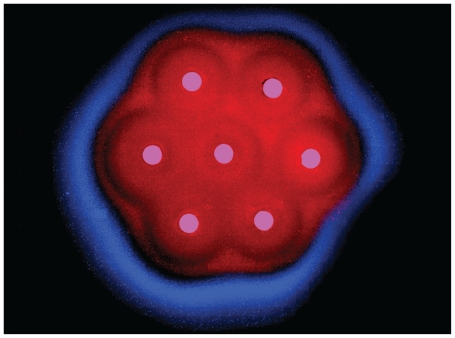
**Above:** Rick Weiss of MIT and colleagues engineered *E. coli* “receiver” cells to evaluate how far they are from pink “sender” cells and report this distance by expressing a particular fluorescent protein (red or blue). The effect somewhat resembles embryogenesis, in which the maternal environment provides such cues using chemical gradients.

**Figure:**
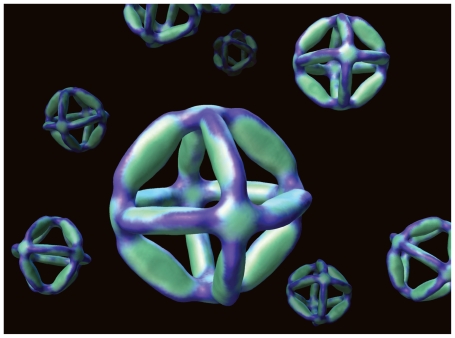
**Below:** William Shih of Harvard and colleagues designed a single DNA strand that folds itself into a nanoscale octahedron using a technique called nano-origami. These minute structures could be used in molecular manufacturing, as structures that ferry drug molecules directly to diseased cells, or in X-ray crystallography.

